# Burn-Induced Acute Kidney Injury–Two-Lane Road: From Molecular to Clinical Aspects

**DOI:** 10.3390/ijms23158712

**Published:** 2022-08-05

**Authors:** Andrei Niculae, Ileana Peride, Mirela Tiglis, Evgeni Sharkov, Tiberiu Paul Neagu, Ioan Lascar, Ionel Alexandru Checherita

**Affiliations:** 1 Clinical Department No. 3, “Carol Davila” University of Medicine and Pharmacy, 050474 Bucharest, Romania; 2 Clinical Department No. 14, “Carol Davila” University of Medicine and Pharmacy, 050474 Bucharest, Romania; 3 “Alexandrovska” University Hospital, 1000 Sofia, Bulgaria; 4 Clinical Department No. 11, “Carol Davila” University of Medicine and Pharmacy, 050474 Bucharest, Romania

**Keywords:** burn, acute kidney injury, NGAL, cystatin C, KIM-1, TIMP-2, IGFBP7

## Abstract

Severe burn injuries lead to acute kidney injury (AKI) development, increasing the mortality risk up to 28–100%. In addition, there is an increase in hospitalization days and complications appearance. Various factors are responsible for acute or late AKI debut, like hypovolemia, important inflammatory response, excessive load of denatured proteins, sepsis, and severe organic dysfunction. The main measure to improve the prognosis of these patients is rapidly recognizing this condition and reversing the underlying events. For this reason, different renal biomarkers have been studied over the years for early identification of burn-induced AKI, like neutrophil gelatinase-associated lipocalin (NGAL), cystatin C, kidney injury molecule-1 (KIM-1), tissue inhibitor of metalloproteinase-2 (TIMP-2), interleukin-18 (IL-18), and insulin-like growth factor-binding protein 7 (IGFBP7). The fundamental purpose of these studies is to find a way to recognize and prevent acute renal injury progression early in order to decrease the risk of mortality and chronic kidney disease (CKD) onset.

## 1. Introduction

Acute kidney injury (AKI) is one of the main complications in patients with severe burn injuries, associated with high mortality levels, therefore being a major health problem, in addition to the burn injury burden [1,2]. It is characterized by an abrupt, rapid, and sustained reduction in renal function [3,4]. In the last decade, a revised classification of acute renal impairment has been proposed, based on the KDIGO (Kidney Disease: Improving Global Outcomes) guidelines, in order to better comprehend the possibility of progression to chronic kidney disease [5]:

- AKI—duration of ≤7 days, presenting an increase of serum creatinine level more than 50% within 7 days or by ≥0.3 mg/dL within 2 days or oliguria more than 4 h; at this point, no structural changes are defined.
- AKD (acute kidney disease or disorders)—duration of ≤3 months, presenting AKI or a glomerular filtration rate (GFR) < 60 mL/min/1.73 m^2^ or a decrease of GFR more than 35% from the baseline value or increase of serum creatinine level of 50% higher than baseline; different structural anomalies can be noted, such as albuminuria, hematuria, pyuria, etc.
- After 3 months of renal impairment, according to the definition, chronic kidney disease (CKD) is considered (GFR < 60 mL/min/1.73 m^2^).

Furthermore, depending on the severity of the renal impairment associated with AKI, a Saxena et al. study, performed on patients with AKI admitted to the intensive care unit, concluded that a higher risk of mortality was observed in patients with AKI stages 2 and 3. The proposed classification of AKI severity included three major stages [6]:

- Stage 1—an increase in serum creatinine level ≥ 0.3 mg/dL or 1.5–1.9 times higher than the baseline value.
- Stage 2—an increase in serum creatinine level more than 2–2.9 times higher than baseline.
- Stage 3—an increase in serum creatinine level ≥ 3 times higher or >4 mg/dL than baseline or the requirement of renal replacement therapy initiation.

In several studies, these findings were also reported in burned patients diagnosed with AKI, noticing that the risk of mortality can be linked to the severity of AKI (based on RIFLE criteria): an increased chance of death, especially in patients evaluated as RIFLE-injury and RIFLE-failure. The RIFLE criteria consist of the following stages [1]:

- Risk (R)—an increase in serum creatinine level of 1.5–1.9 times higher than the baseline value or a decrease of GFR > 25% or a urine output < 0.5 mL/body weight/hour within 6–12 h.
- Injury (I)—an increase in serum creatinine level of 2–2.9 times higher than baseline or a decrease of GFR > 50% or a urine output < 0.5 mL/body weight/hour within 12 h.
- Failure (F)—an increase in serum creatinine level of 3 times higher or >4 mg/dL than baseline or a decrease of GFR > 75% or a urine output < 0.5 mL/body weight/hour within 24 h or anuria notice more than 24 h.
- Loss (L)—the loss of renal function > 4 weeks.
- End Stage (E)—the loss of renal function > 3 months.

Until recently, it was considered that early development of burned-induced AKI is associated with negative short-time outcomes regarding not only mortality but also morbidity [7]. Lately, it appears that late-onset AKI leads to higher 28-day and 90-day mortality rates [8].

It is thought to affect about a third of the burn population [4], with mortality ranging from 28% to 100% in severe cases [9,10,11]. A study published by Clark et al., which evaluated 1040 patients with thermal burns, showed that injuries affecting ≤ 40% of the total body surface area (TBSA) lead to AKI stage 1 development, while in patients with extensive-area burns, ≥40% TBSA, could evolve to severe forms [12]. In addition, AKI is independently associated with an increase in length of hospital stay (LOS) and in-hospital mortality [13,14].

Classically, burn-related AKI can develop early (up to three days after burn incident), or late (starting from day four after injury) during hospitalization. The etiology of early post-burn AKI includes hypovolemia due to important fluid loss, increased inflammatory mediators and denatured protein release, cardiac dysfunction, rhabdomyolysis, etc. [7,15,16,17,18]. Late acute renal failure occurs especially in the context of sepsis and multi-organ dysfunction syndrome (MODS), or due to fluid overload, and nephrotoxic usage [7,9,12,19,20,21].

Creatinine, a molecule intensively used both for the diagnosis of renal failure and for monitoring renal function, has been shown not to be an accurate reflection of acute changes, with plasma values increasing only when GFR decreases by 30–40% [2,22,23,24]. Further, various elements, such as demographics (age, sex, and ethnicity), weight, catabolic state, or concomitant use of certain drugs can influence serum creatinine trends [2], with a half-life of around 4 h [23,25]. In burn patients, the situation is much more complicated. Prerenal azotemia can develop due to inappropriate fluid resuscitation, with consecutive dehydration and hypovolemia, or in the late stage of evolution, as a result of the septic shock appearance, creatinine generation can be reduced or can have false negative values in the presence of fluid overload [26,27].

Several studies have focused on the problem of identifying certain markers that allow early diagnosis and management of acute renal failure in burn patients, like neutrophil gelatinase-associated lipocalin (NGAL), cystatin C, kidney injury molecule-1 (KIM-1), tissue inhibitor of metalloproteinase-2 (TIMP-2), interleukin-18 (IL-18), and insulin-like growth factor-binding protein 7 (IGFBP7) [28,29,30,31]. Future research is needed to analyze and standardize some of these biomarkers in critical burn patients and establish cut-off values.

It is well known that even after an episode of AKI, the risk of developing CKD is high [32,33], with an important impact on patients’ quality of life, in addition to the functional and aesthetic sequela of post-combustion injuries. Therefore, the primary purpose of this narrative review is to identify the relevant biomarkers in burn-induced AKI prediction in order to increase the survival of this subgroup of patients.

## 2. Etiology and Risk Factors of Acute Kidney Injury in Burn Patients

Even though the exact etiology of AKI development in patients with burn lesions is not clear, various researchers state that it is most likely multifactorial [34]. Identification of etiological and risk factors helps clinicians improve burn patients’ prognostic and guide therapeutic management, being crucial during entire hospitalization. Rakkolainen et al. showed that even a small increase in serum creatinine value has a severe impact on critically burned patients’ survival [35].

**Figure 1:**
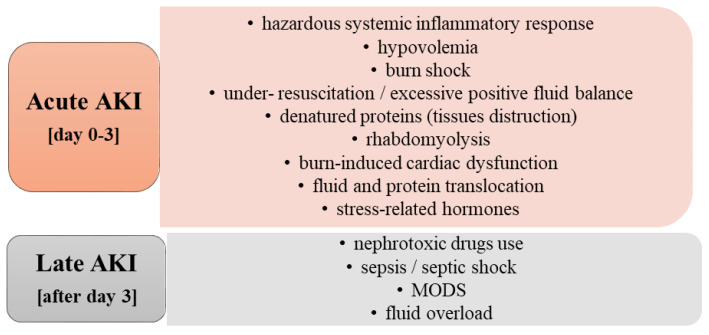
Etiology of burn-induced acute kidney injury. Notes: AKI—acute kidney injury; MODS—multiple organ dysfunction syndrome.

As we emphasized before, for better understanding and differentiation, burn-induced AKI is often classified as having an acute or late appearance [7]. The main incriminated etiological factors are presented in Figure 1 [7,8,9,15,16,17,18,19,20,21,27,36,37,38,39,40].

### 2.1. Acute AKI

In face of decreased circulatory volume (fluid shift, under-resuscitation, important fluid loss), the tissue perfusion is reduced (hypoperfusion) with consecutive reduction of the glomerular filtration rate (GFR), and secondary hypoxia leads to irreversible ischemia and tubular necrosis [41,42,43]. It is well known that the pathophysiological mechanisms incriminated in the development of acute tubular necrosis are triggered by ischemia or nephrotoxins. Further on, the following abnormalities can be noticed, responsible for the decrease of GFR and, consequently, the urine output, and, nevertheless, the progression of renal impairment [44]:

▪ Hemodynamic changes,
  - Abnormal renal autoregulation—in cases of a systolic blood pressure value < 80 mmHg or due to an insult that interferes with prostaglandins activity,
  - Intrarenal vasoconstriction and the injury of the endothelial cells—an endothelial impairment is noticed that contributes to the decrease of nitric oxide and prostaglandins, the increase of endothelin, and the swelling of endothelial cells, and also an important synthesis of adhesion molecules (such as intracellular adhesion molecule 1). These changes determine significant intrarenal vasoconstriction, abnormal autoregulation, hypoxia, increased synthesis of reactive oxygen species, with a direct impact on the drop fall of GFR,
  - Tubuloglomerular feedback—it could present a beneficial role as there is a limitation in delivering sodium to the impaired tubules, once the GFR decreases.
▪ Injury of the tubular epithelial cells
  - Cell death—it occurs only in a few renal tubules, especially due to apoptosis, not necrosis,
  - Disruption of actin cytoskeleton—it is responsible for the loss of polarity that, in the proximal convoluted tubule, contributes to impaired sodium reabsorption and, consequently, elevated distal sodium chloride, which represents the trigger for the onset of tubuloglomerular feedback. Furthermore, actin cytoskeleton disruption determines cell detachment and decreased cell-matrix adhesion leading to an accumulation of tubular cells in the tubules; consequently, cast formation and intratubular obstruction are emphasized,
  - Backleak—its onset is linked to the loss of adhesion molecules and junction proteins, leading to the leak back of the filtrate into the renal interstitium, favoring also interstitial inflammation. This abnormality is especially noticed in severe forms of acute tubular necrosis.
▪ Inflammatory state—due to the presence of ischemia, different pro-inflammatory immune mechanisms are activated
  - High synthesis of toll-like receptors 2 and 4 that contribute to chemokines release,
  - Activation of the alternative pathway of the complement that favors the synthesis of cytokines (such as interleukin 6, tumor necrosis factor-α, etc.) and chemokines, contributing to the increase of leukocytes and a direct vasoactive effect,
  - Neutrophil activation along with the presence of reactive species of oxygen and proteases will lead to an increased renal injury. Similarly, the activation of monocytes will exacerbate the severity of the injury,
  - Additionally, T and B cell upregulation can contribute to the extension of renal impairment.

All of these also contribute to the excessive burn-induced systemic inflammatory response [45,46,47]. Therefore, early AKI increases the hypermetabolic state of the burned patient [46].

It is common knowledge that fluid resuscitation represents the fundamental element of burn patients’ management. In the last years, the problem of over-resuscitation has been intensively studied due to various complications with a major impact on mortality. It has been noted that positive fluid balance is incriminated in the onset of intraabdominal hypertension and abdominal compartment syndrome which increase the risk of acute renal injury (a mechanism similar to that in fluid overload) [7,9,13,21,27,48].

In fact, in burned patients, the ischemic insult (determined by the decreased renal blood flow) induces the activation of oxygen free radicals, which are responsible for renal tubular injury and the damage of the tubular cellular junctions. These anomalies contribute to casts development, and further on to tubular obstruction and urine back-flow, resulting in an even lower GFR that contributes to the onset and progression of acute renal impairment [7].

Another important factor incriminated in the development of burn-induced AKI is the cardiac dysfunction that presumably is caused by [7]:

▪ Sympathetic overactivity simultaneous with an insufficient response of the adrenal gland,
▪ Hypovolemia that leads to important myocardial ischemia,
▪ Tumor necrosis factor-alpha (TNF-α) activation, which has a direct impact on myocardial suppression; in the presence of endotoxins or thermal injury, the myocytes synthesize TNF-α, which contributes to an impaired response to catecholamine, to a low ejection fraction, and even to the presence of biventricular dilatation.

Rhabdomyolysis is another key feature responsible for the onset and progression of burn-induced AKI, a factor precipitated by muscle damage consequently to thermal or electrical injury and even to the development of compartment syndrome [7]. In this case, myoglobin is released into the systemic circulation and precipitates in the renal tubules (myoglobinuria) leading to afferent renal arteriolar vasoconstriction along with oxygen free radicals’ activation; myoglobinuria induces ischemic tubular injury in the proximal tubules and tubular obstruction in the distal tubules, contributing, in this manner to the onset of AKI [7].

### 2.2. Late AKI

Five possible pathways to explain the influence of fluid overload in AKI onset have been described [7,9,13,21,27,49]:

▪ Intraabdominal hypertension, defined as an increased intraabdominal pressure over 12 mm Hg, represents an important risk factor. In face of intraabdominal hypertension >20 mmHg (considered the value mostly associated with organ dysfunction onset) and abdominal syndrome development, the renal perfusion is decreased inducing reduced GFR, which along with inflammatory cytokines and denatured proteins, contributes to burn-induced AKI progression;
▪ Interstitial edema leads to increased interstitial pressure, altered renal oxygenation, and impaired cellular junctions; the kidneys’ response to this new high pressure is inadequate due to the renal capsule limitation. All these disturbances produced by the presence of interstitial edema are contributing to the onset of renal congestion, decreased renal perfusion, and a significant lowered GFR, which finally determine the development of AKI;
▪ Endothelial dysfunction produces glycocalyx impairment and capillary leakage that determine interstitial edema, and reduced systemic intravascular volume, leading to decreased renal perfusion and subsequently to AKI;
▪ Atrial natriuretic peptide (ANP) is synthetized due to hypervolemia that leads to the stretching of atria and blood vessels. The presence of ANP contributes to the impairment of glycocalyx and further on the capillary leakage that, as already mentioned, are incriminated in the development of AKI;
▪ Bowel wall edema favors the access of bacteria into the systemic vascular circulation, leading to sepsis that represents an important cause of AKI by altering the endothelial function.

Nevertheless, the major causes of burn-induced AKI, especially for late onset, are represented by the use of nephrotoxic drugs, and by sepsis that is precipitated mainly by systemic arterial vasodilatation as a consequence of decreased vascular resistance. This high-flow/low-flow condition is determined by the presence of bacteria that induces a cytokines response with a direct impact on the onset of endothelial injury (as already mentioned), procoagulant state, and vasoparalysis, contributing to excessive hypotension. To contra-balance this hypotension state, the cardiac output increases due to the activation of sympathetic and renin-angiotensin-aldosterone systems. Furthermore, during sepsis, significant tubular inflammation and microvascular insult are noted, which determine high tubular pressure and afferent renal arteriolar vasoconstriction leading to a further decrease in GFR [7].

In summary, during burn injuries, an important vascular permeability is noted, that permits the passage of large oncotic molecules from the vessels into the tissues leading to decreased vessel oncotic pressure and circulating blood volume, and also explaining the presence of important swelling of the burned areas; the final effect is the decrease of blood flow in vital organs, including the kidneys. It should be emphasized that, except for this capillary leakage, the decrease of systemic blood volume is influenced also by water evaporation in burned sites [35]. All these pathophysiological mechanisms involved in the onset of burn-induced AKI support the idea that burned individuals represent a particular group of patients that should benefit from intensive and adequate treatment and assessment management.

Therefore, special attention should be focused on the clinical characteristics of this group of patients in order to decrease the onset and severity of burn-induced AKI. A recent study by Chen et al. evaluated the main clinical characteristics and risk factors incriminated in the development of early AKI in severely burned patients and observed that rhabdomyolysis, TBSA, full-thickness injuries, and ABSI (the abbreviated burn severity index) are independent risk factors responsible for AKI early development. While electrical injuries, full-thickness TBSA, and rhabdomyolysis can determine severe forms of early AKI. The study’s conclusion was that optimal management of rhabdomyolysis might lead to a better outcome, reducing the risk of early and severe forms of AKI development [50].

The key to treatment in such instances remains the reversal of the underlying event [7,51]. Precocious decompression escharotomy reduces the risk of AKI development, especially during the late phase of evolution [6]. Regarding the use of renal replacement therapy (RRT) to improve survival and reduce chronic kidney injuries, studies are scarce in terms of optimal timing and methods, aimed more at an early approach [52,53].

**Figure 2:**
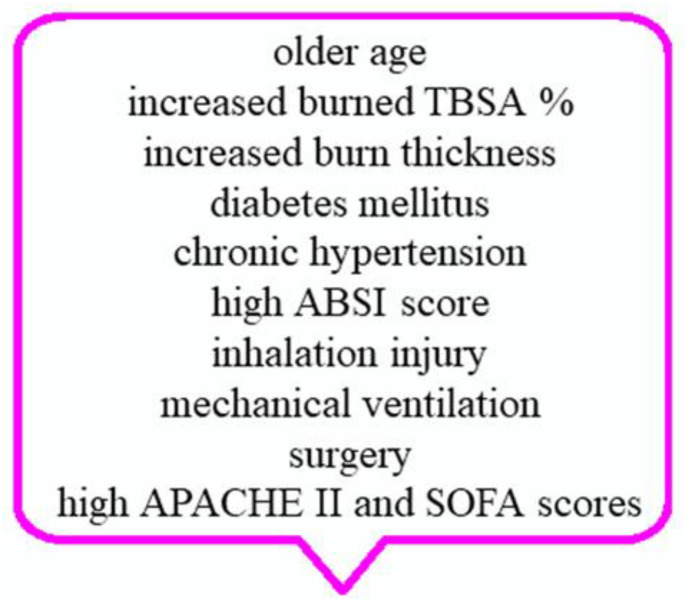
Main risk factors of burned-induced AKI. Notes: ABSI—the abbreviated burn severity index; APACHE II—acute physiology and chronic health evaluation II; SOFA—sequential organ failure assessment; TBSA—total body surface area.

Considering all the mechanisms involved in the onset of AKI, there are various research studies aimed at identifying risk factors for the development of AKI in severe burns, as shown in Figure 2 [13,17,35,38]. Additional risk factors are represented by flame injury, tracheotomy, pre-existing coronary disease, congestive heart failure, liver failure, intraabdominal hypertension or abdominal compartment syndrome, catheter infection, and increased levels of creatinine and urea nitrogen at admission [8,10,13,17,54].

## 3. Particular Biomarkers of Renal Injury

Until the discovery of novel biomarkers of renal injury, the assessment of AKI was exclusively based on serum creatinine levels and urine output. Considering that serum creatinine concentration could be influenced by numerous factors, including an important muscle loss observed especially in critically ill patients, and that a decrease in urine output can be noticed, as already mentioned above, after the onset of different AKI insults, the need for early detection of AKI onset has gained more interest. Furthermore, several studies showed that serum creatinine concentration and urine output cannot represent viable tools in evaluating the risk of AKI in burned patients [55]. Nevertheless, even if recently different biomarkers have been identified for a timely diagnosis of AKI, their general practical applications are still missing, considering that the current AKI classification does not include them, and, additionally, there is a limitation in the possibility of rapidly assessing them in general practice. Recently, an integrative model of these biomarkers and their corresponding role in determining the risk of AKI has been proposed, similar to the arterial blood gas assessment used in cardiopulmonary stability [56]:

- For the assessment of homeostasis (including the required volume repletion)—urine output,
- For the evaluation of glomerular filtration—serum creatinine,
- For the assessment of tubular injury—NGAL (neutrophil gelatinase-associated lipocalin),
- For determining the renal function reserve—furosemide stress test and measured renal functional reserve (using high oral protein intake to evaluate renal response to this significant protein load; GFR was measured before and after the intake),
- For monitoring the system stress—TIMP-2 (tissue inhibitor of metalloproteinase-2) and IGFBP7 (insulin-like growth factor-binding protein 7).

Furthermore, according to Wasung et al., there are a few characteristics that the “ideal” biomarker for renal dysfunction should meet, like being non-invasive, site-specific, with high sensitivity and specificity in correlating with the burden of renal injury, to be able to rapidly and accurately increase in response to kidney disease. It is also important not to be influenced by different populations and not to interfere with usual drugs, to have the ability to provide risk stratification in order to make a prognosis, and last but not least, to be a stable molecule at different temperatures and pH [22]. Other required aspects are represented by the capability of increasing urine rapidly after a renal injury, to remain at high levels during the entire episode and to decrease over the recovery period, and to quantitatively reflect the intensity of the injury [57].

### 3.1. Neutrophil Gelatinase-Associated Lipocalin (NGAL)

NGAL, part of the lipocalin family, is a molecule that originates from neutrophils and epithelial cells (kidney, intestine, lungs, and trachea), which can be secreted in the dimeric or monomeric form, the latter being specific only to the kidney [58,59,60,61]. It has a molecular weight of 25 kDa and is linked to gelatinase from neutrophils [62,63], which can be rapidly measured in plasma or urine through enzyme-linked immunosorbent assay (ELISA) [64]. After release, by cause of a pre-renal event (hypotension, heart failure, sepsis/septic shock), NGAL is secreted into the glomeruli and then reabsorbed into the proximal tubules [63,65]. Commonly, low levels are found in the bloodstream, 40–100 ng/mL, due to free filtration and a short half-life (around 10 min). Under pathological conditions, serum NGAL rapidly rises due to increased secretion and decreased GFR, values ≥ 155 nmol/L having high specificity and sensitivity for AKI diagnosis. Urine NGAL levels increase within 3 h after injury, with a peak after 6 h [58,66,67,68]. Furthermore, a recent study noticed that NGAL could represent an important biomarker in assessing the risk of AKI in burned patients [55]. It should be emphasized that the renal source of NGAL mRNA is detected in the distal nephron, while the proximal convoluted tubule (PCT) is responsible for its reabsorption, raising the possibility of representing also a biomarker for PCT insult, but the major source of urine NGAL remains the impairment of distal nephron [69].

### 3.2. Cystatin C

Cystatin C is an endogenous proteinase inhibitor, produced continuously by the nucleated cells, with a molecular weight of 133 kDa and a half-life of 90–120 min [70,71]. It is not affected by age, gender, or muscle mass like creatinine, is filtered by kidney glomerular cells, reabsorbed almost entirely in the PCT, and then catabolized by epithelial cells at this level. Only a small fraction is excreted through urine [72,73]. Therefore, serum changes of cystatin C appear before creatinine modification, in 3–6 h after a renal insult, with a peak at 48 h [57,71]. In addition, Cystatin C levels can be influenced by systemic inflammation, especially for patients with major burns at risk of developing rapid infections [73].

### 3.3. Kidney Injury Molecule-1 (KIM-1)

KIM-1 is a type 1 transmembrane protein, released by tubular epithelium cells in the face of various injuries, with a molecular weight of 38.7 kDa [74,75]. It consists of extracellular domains of mucin and immunoglobulin. KIM-1 action is up-regulated in proximal tubular epithelial cells [22]. Basal expression is very low in normal kidneys, but it is regulated 48 h after ischemia-perfusion injury through the proliferation of epithelial cells of PCT [76]. It is a marker of both kidney injury and repair [77,78], starting to increase at 6 h after injury, with a peak value at about 48 h [79]. Furthermore, according to recent studies, KIM-1 could represent a better predictive tool in assessing AKI in lung-cancer patients, presenting a more increased concentration compared to NGAL [80].

### 3.4. Tissue Inhibitor of Metalloproteinase-2 (TIMP-2) and Insulin-like Growth Factor-Binding Protein 7 (IGFBP7)

TIMP-2 and IGFBP7, expressed and secreted in the tubular cells, are markers of G1 cell cycle arrest, playing an important role during the early phase of cellular stress, the first having a molecular weight of 24 kDa, and the second of 29 kDa. They block stage G1 of the tubular cell cycle and appear to have a reno-protective role [81,82,83,84]. They are considered to represent stress biomarkers [30]. Usually, TIMP-2 is secreted by cells of the distal tubules, while IGFBP7 by PCT cells [85]. In patients with AKI, urinary TIMP-2/IGFBP7, they appear to anticipate the need for renal replacement therapy (RRT), to predict the outcome, kidney recovery, and the development or progression of CKD [30,86,87]. These biomarkers can forecast the appearance of moderate and severe AKI in high-risk patients within 12 h [82]. TIMP-2 and IGFBP7 are considered biomarkers of the pre-injury phase, as they are filtrated by glomeruli [69,88]. In addition, there is evidence that TIMP-2 and IGFBP7 can better predict the development of moderate and severe stages of AKI. Although the majority of data concluded that NGAL (tubular biomarker) represents the first choice for the early detection of AKI, Sakyi et al. study showed that a combination between the stress biomarkers and the tubular one could represent a second-best tool in predicting the onset of moderate to severe stages of AKI [30].

### 3.5. Interleukin-18 (IL-18)

It is a proinflammatory biomarker, part of the IL-1 superfamily, with a 22 kDa molecular weight, which promotes endogenous inflammatory processes [89,90,91]. Studies have shown that IL-18 levels increase within 24–48 h before AKI development (ischemia-reperfusion injury especially), with about a median of 2 days ahead of serum creatinine or urea nitrogen modifications [90,92,93]. Urinary IL-18 has high sensitivity and specificity (more than 90%) for AKI diagnosis [88,94,95]. Urine levels of IL-18 increase during the first 6 h after kidney injury, with a peak after 12–18 h [96]. It should be highlighted that several studies concluded that IL-18 can represent a useful biomarker in detecting especially acute tubular necrosis, rather than pre-renal AKI, but as it could be increased also in septic patients (associated complication often detected in burned patients), the interpretation of this elevation should be performed with caution. In addition, compared to NGAL levels, IL-18 presents a slower increase [97].

**Figure 3:**
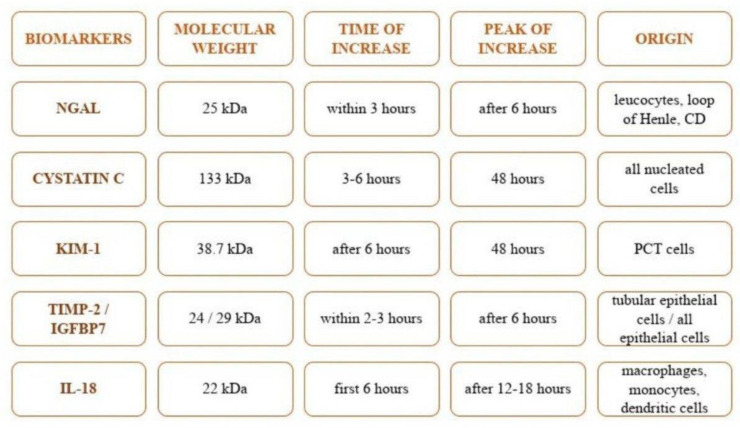
The main feature of usual biomarkers used in burn-induced AKI diagnosis. Notes: Levels are measured in urine. NGAL—neutrophil gelatinase-associated lipocalin; KIM-1—kidney injury molecule-1; TIMP-2—tissue inhibitor of metalloproteinase-2; IGFBP7—insulin-like growth factor-binding protein; IL-18—interleukin-18; PCT—proximal convoluted tubule, CD—collecting duct.

**Figure 4:**
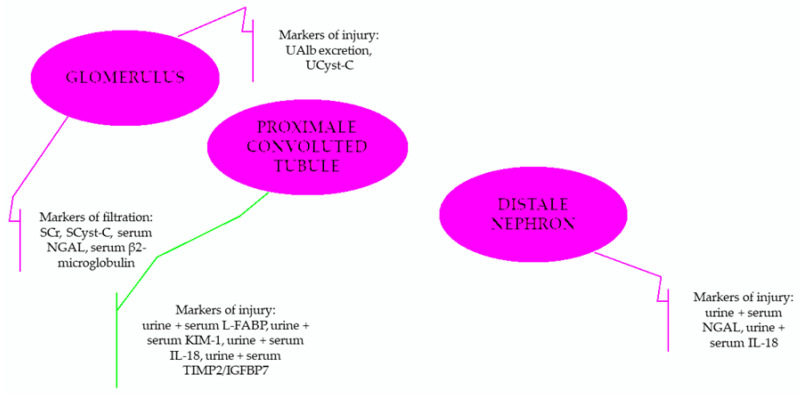
The role of novel biomarkers in identifying the site of AKI insult. Notes: IGFBP7 = insulin-like growth factor-binding protein 7; IL-18 = interleukin-18; KIM-1 = kidney injury molecule-1; L-FABP = liver-type fatty acid-binding protein; NGAL = neutrophil gelatinase-associated lipocalin; SCr = serum creatinine; SCyst-C = serum cystatin-C; TIMP2 = tissue inhibitor of metalloproteinase-2; UAlb = urine albumin; UCyst-C = urine cystatin-C (Modified after [69,101]).

To summarize, the main features and renal localization of usual biomarkers used for burn-induced AKI diagnosis are presented in Figure 3 and Figure 4. Normally, after a kidney insult, IL-18 induces additional injuries during the inflammatory phase. NGAL, through its antiapoptotic properties, reduces this extensive response, with TIMP-2 and IGFBP7 attenuating further the renal injury (there are some reports about IGFBP7 role in promoting injury). KIM-1 and TIMP-2 appear to support renal tissue recovery and remodeling, with NGAL stimulating tubular cell proliferation [69,78,98,99,100,101]. Furthermore, it has been documented that the combined assessment of several biomarkers could better predict the development and evolution of AKI [69]:

- Serum creatinine + NGAL—can predict the risk of mortality, lengths of hospital stay, the need for intensive care management, and also renal replacement therapy requirement.
- Serum creatinine + NGAL + KIM-1—predict better the need of renal replacement therapy and the risk of mortality within 7 days form the onset of AKI comparing with only the evaluation of serum creatinine.
- NGAL + Cyst-C—highlight the risk of severe forms of AKI onset.

Functional biomarkers (such as Cyst-C)—can predict transient forms of AKI.

## 4. Clinical Use of Particular Biomarkers in Burn-Induced AKI

Over the last years, various biomarkers have shown utility in predicting the risk of AKI development in burn patients, superior to well-known and utilized tools, like hourly urine output (UO), serum creatinine, and blood urea nitrogen (BUN) measurements.

In face of severe burn injuries, the risk of AKI development is high, as we emphasized before. At the same time, AKI has not only a negative impact on the short outcome of these patients but also increases long-term complications, like CKD or end-stage kidney disease (ESKD) [102,103], especially in fragile individuals [104]. Therefore, the burden and strain on the health care systems are extremely high when these two entities coexist [105,106].

As presented, the use of KDIGO or RIFLE criteria is useful in burn-induced AKI diagnosis, along with the revision of previous medical conditions, treatments, and physical examination, but not enough [1,5,107]. Nevertheless, in patients with severe burn injuries, this diagnosis is not easy, considering that clinically, the UO can be relatively normal, and due to important fluid resuscitation in many cases, even under-appreciated, and the serum creatinine levels do not rapidly increase despite renal injury appearance [7]. UO, even though it is a specific sign of burn-induced kidney injury development, it is not sensitive with respect to AKI diagnosis [108]. The use of usual urine chemical markers may help diagnose, evaluate and identify the underlying cause of AKI [109,110]. Therefore, taking into consideration the limitation of these two main elements in rapid early AKI diagnosis, the so-called subclinical-AKI, the novel biomarkers are of paramount importance in face of signs and symptoms absence [107].

In critically ill patients, Shoaib et al. showed that urine NGAL has high accuracy and sensitivity in diagnosing early AKI, with around 24–48 h before serum creatinine rises, along with the ability to predict the need for renal replacement therapy (RRT), outcome and to identify prerenal azotemia [111]. Sen et al. presented that the whole blood NGAL is an independent predictor of AKI development in the first four hours after severe burn injury compared to serum creatinine or UO, which showed no significant changes, nor had predictive values, in patients who developed AKI in the first week after admission [67]. Urine NGAL also shows good results in predicting renal injury occurrence starting with the fourth week of burn evolution, as demonstrated in a study including 84 patients with severe burns [71].

Another study reports good results regarding plasma NGAL capability of predicting AKI in burn patients, and at the same time anticipating morbidity and mortality in these patients [58]. In this regard, a report by Dépret et al., which targeted 87 patients, presented that plasma NGAL is a good predictor of severe burn trauma [11].

The same trend continued in a study published by Yang et al., which showed that high levels of plasma and urine NGAL are predictive of early burn-induced AKI and mortality in patients developing burn shock, without being a useful indicator for late AKI [18]. However, in this cohort of 19 patients with critical burns, even though cystatin C level increased in the first 12 h after injury and was independently associated with AKI appearance, it has not exhibited superiority to NGAL, having similar results as serum creatinine [64].

A study including 258 severe burn patients, published by Emami et al., emphasized the importance of identifying prognostic biomarkers in rapid AKI diagnosis due to increased hospital length of stay (LOS) and excessive mortality rates. At the same time, according to previous reports, creatinine has been shown not to be a practical prognostic element, but there was an association identified between BUN and burn-induced AKI [112].

Nevertheless, things are more complicated in the severe burn patient. Chun et al. showed that serum NGAL increases within 7 days before burn-induced AKI development, being significantly correlated with TBSA, AKI, and mortality. NGAL is both a reflection of AKI appearance and an important inflammatory state in burn patients. Therefore, it should not be used as a single marker in order to predict AKI in this population [28].

Reports regarding cystatin C utility in burn-induced AKI are scarce. Yim et al. revealed, in a study including 97 patients with major burns, that serum cystatin C is a useful marker in AKI diagnosis [29]. It appears to be superior to serum creatinine for critically ill patients in predicting renal outcomes [113,114]. Cystatin C has superior accuracy and sensitivity for AKI identification after critical burns, especially in patients with older age and large TBSA [115].

Urinary KIM-1 is in high levels in patients with severe burn injuries, detected earlier compared to serum creatinine. It also shows the ability and sensitivity to predict AKI development upon admission, correlating with TBSA, APACHE II score, and rhabdomyolysis presence. Furthermore, KIM-1 and IL-18 can be used to predict AKI development in the post-burn period [89,116]. Further studies in this field are required in order to show the usefulness of this molecule in severe burn patients.

Studies regarding urinary TIMP-2 and IGFBP7 utility in burn patients are limited. A recent report from a Trauma Center showed that these markers are superior in early prediction and diagnosis of AKI in high-risk patients. The utility of NGAL in combination with IGFBP7 appears to be the second-best choice, with NGAL as a singular biomarker being the least predictive [30]. A meta-analysis addressing the same issue demonstrated that these urinary biomarkers are useful, especially in ruling-out AKI diagnosis [86]. Over the years, these two urinary markers showed utility in AKI development following cardiac and major surgery, severe trauma, kidney transplant, decompensated heart failure, cardiac arrest, sepsis, and toxic renal disease [117,118,119,120].

## 5. Conclusions

Major burns are complex trauma requiring a multidisciplinary approach. Burned-induced AKI, which affects around a third of the severe burn population, is a major health problem, with a negative impact on morbidity and mortality in this subgroup of patients. Even though, after an AKI episode, most patients recover their kidney function, the risk of CKD development remains high. Particular serum and urinary biomarkers have shown their utility in AKI prediction in critically ill patients, and various studies reported their efficacy in patients with burn injuries. Among these, serum and urine NGAL, urinary TIMP-2, and IGFBP7 have superior results. After these, the following important biomarker seems to be cystatin C and urinary KIM-1, but further studies are required in order to validate such markers in burn patients and establish cut-off values.

## Data Availability

Not applicable.
